# FDMLNet: A Frequency-Division and Multiscale Learning Network for Enhancing Low-Light Image

**DOI:** 10.3390/s22218244

**Published:** 2022-10-27

**Authors:** Haoxiang Lu, Junming Gong, Zhenbing Liu, Rushi Lan, Xipeng Pan

**Affiliations:** School of Computer and Information Security, Guilin University of Electronic Technology, Guilin 541004, China

**Keywords:** low-light image enhancement, guided filter, multiscale representation, attention mechanism

## Abstract

Low-illumination images exhibit low brightness, blurry details, and color casts, which present us an unnatural visual experience and further have a negative effect on other visual applications. Data-driven approaches show tremendous potential for lighting up the image brightness while preserving its visual naturalness. However, these methods introduce hand-crafted holes and noise enlargement or over/under enhancement and color deviation. For mitigating these challenging issues, this paper presents a frequency division and multiscale learning network named FDMLNet, including two subnets, DetNet and StruNet. This design first applies the guided filter to separate the high and low frequencies of authentic images, then DetNet and StruNet are, respectively, developed to process them, to fully explore their information at different frequencies. In StruNet, a feasible feature extraction module (FFEM), grouped by multiscale learning block (MSL) and a dual-branch channel attention mechanism (DCAM), is injected to promote its multiscale representation ability. In addition, three FFEMs are connected in a new dense connectivity meant to utilize multilevel features. Extensive quantitative and qualitative experiments on public benchmarks demonstrate that our FDMLNet outperforms state-of-the-art approaches benefiting from its stronger multiscale feature expression and extraction ability.

## 1. Introduction

Photos captured in insufficient illumination conditions such as nighttime, lopsided, under-exposed, etc., exhibit an undesired visual experience or deliver compromised messages for other computer vision tasks, due to their low contrast and lightness and blurry details [1,2,3,4,5]. Especially, high-level computer vision tasks show unsatisfactory performance in these low-light photos, such as in inaccurate face or object recognition [6,7]. Hence, it is necessary to restore the quality of low-illumination pictures. Low-light image enhancement (LLIE) [1,8,9,10,11,12,13,14] is an efficient way to yield visually pleasing images with moderate lightness, vivid color, and clearer details, so as to further improve the performance of face detection, object recognition, and other tasks. Therefore, LLIE [1,2,3,15] is an indispensable technology in low-level computer vision applications to generate wanted images.

In past decades, a great deal of LLIE approaches, including histogram-based [3,16,17], Retinex-based [8,9,10,18,19], fusion-based [20,21], physical-model-based, [3,22,23,24,25,26] have been reported. Histogram-based methods, which are simple and highly efficient, introduce an over- or underenhancement owing to the spatial relationship among pixels being neglected. Retinex-based methods consider that an image consists of illumination and reflection components, and the enhanced images exhibit color distortion. Fusion-based models yield appealing visual images, benefiting from fusing multiple images with various characteristics. However, the enhanced results encounter a detail loss and artificial halos. Dehazing model-based approaches [25] are the most typical representative of physical-model-based methods, and they are unsuccessful for creating satisfying and hazy-free images. Recently, data-driven methods [1,27,28,29,30] have been introduced to conquer the inappropriate enhancement of classical methods, owing to their powerful feature extraction capability. However, existing approaches are confronted with heavy computing burdens and are time-consuming, limiting their real-world applications. Furthermore, most of them rarely take hierarchical features and a multiscale representation into account [15].

**Figure 1:**
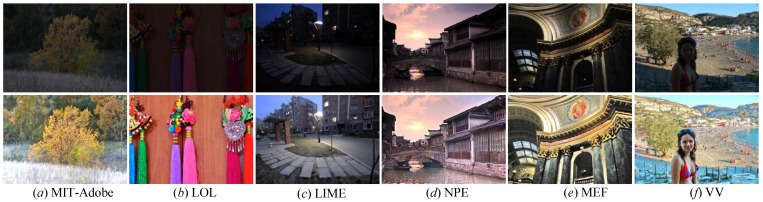
Samples of the presented images (**bottom**) for various images captured under different scenarios (**top**). From left to right, these authentic images are selected from the MIT-Adobe, LOL, LIME, NPE, MEF, and VV benchmarks, respectively.

To cope with these mentioned issues, we propose a new LLIE method based on frequency division and multiscale learning, called FEMLNet, for improving the quality of image acquired in suboptimal lighting conditions. Differing from most CNN-based and GAN-based methods, we perform different operations on the image’s high and low frequencies rather than the whole picture to fully explore its hierarchical features. Additionally, we present a feasible feature extraction module (FFEM) based on a multiscale learning (MSL) block with a dual-branch channel attention mechanism (DCAM) to obtain self-adapting multiscale features. The former can adaptively exploit information at different scale spaces, and the latter makes the focus of our FDMLNet model on more valuable features while enhancing its multiscale learning capacity. Simultaneously, a dense connection strategy is introduced in our model to merge multilevel features adequately. Figure 1 shows the enhanced results via the presented method for the images obtained in different lighting conditions. With the help of our FDMLNet, all enhanced images consistently show a pleasing visual appearance.

In conclusion, our primary contributions of this work are emphasized as follows.

(1) We present a novel LLIE approach for creating visually satisfying images. The superior performance of this FDMLNet is verified by extensive experiments validated on several public benchmarks.
(2) We design a residual multiscale structure named MSAM, which is based on a residual multiscale learning (MSL) block and a dual-branch channel attention mechanism (DCAM). Furthermore, the former promotes the multiscale features learning ability of the FDMLNet, and the latter, including spatial attention and pixels attention, makes our model focus on areas that best characterize the image.
(3) Finally, we merge three MSAMs in a novel dense skip-connection way to build an FFEM for fully exploring the image’s hierarchical information. In addition, we apply the dense connection strategy among FFEMs to further integrate multilevel features adequately.

We organize the rest of this paper as follows. The relevant works on LLIE are briefly reviewed in Section 2. In Section 3, the framework of our model is elaborated. We also present the relation between existing models and our method. In Section 4, we analyze ablation studies and the performance of our FDMLNet in detail. In the end, we report the conclusions and discussions about this work in Section 5.

## 2. Related Works

LLIE plays an irreplaceable role in recovering inherent color and details as well as compressing the noise of low-illumination images. In what follows, we comprehensively review previous low-light image enhancement works, including conventional approaches and leaning-based approaches.

### 2.1. Traditional Approaches

In the early stage, specialized high-performance hardware, such as professional low-light circuits, charge-coupled device (CCD), complementary metal–oxide–semiconductor (CMOS), etc., is employed in imaging systems for generating visually satisfying pictures. However, the price of these devices is unacceptable, and their operation is difficult. We also can process the gathered images by LLIE methods. Histogram-equalization-based methods, including global histogram equalization (GHE) [16,17] and local histogram equalization (LHE) [3,4,5], directly adjust the image pixels value to redistribute their distribution in global and local levels. Swarm intelligence algorithms, image decomposition, Rayleigh distribution, and other technologies [31,32,33] were hired to optimize the previous HE-based approaches. Additionally, gamma, S-shape, logarithmic, and other improved nonlinear functions [34,35,36] also can restore inherent color and details of excessively dark images through pixel transformation. Unfortunately, these above-listed methods either amplify noise or yield improper exposure. Recently, some scholars [37,38,39,40] have handled LLIE issues in the wavelet domain, gradient domain, NSST domain, etc. rather than the spatial domain.

Contrary to pixel transformation approaches, Retinex-inspired methods [8,18,19] typically assume that an image consists of illumination and reflection components, as well as its reflection components’ own consistent peculiarity during the processing. Hence, the LLIE problem can be viewed as the illumination component estimation. On the basis of this assumption, LR3M [18], a fast Retinex-based algorithm [8], Poisson noise aware Retinex model [9], Retinex-based variational framework [10], and other methods [11,41], have been reported to yield satisfying images. However, the enhanced results exhibit observable color distortion, noise enlargement, or fuzzy details. Differing from the above approaches, physical-model-based approaches enhance low-light images from the aspects of the imaging principle. The dehazing model [25], atmospheric scattering model [22,24], and prior-knowledge-based model [23,26] are its typical representative. However, the processed images suffer from hand-crafted halos and local darkness, due to inappropriate prior information under some low-light conditions. Moreover, fusion-based methods [3,20,21], fusing a variety of frequency images or multifeature maps to fully exploit the hierarchical features of the image, can also effectively recover visually satisfactory photos from subpar illumination images. Similar to these, we perform frequency division on low-luminosity images to obtain high- and low-frequency information, and then integrating the frequency images processed by different operations.

### 2.2. Learning-Based Approaches

In recent years, learning-based methods containing supervised and unsupervised learning strategies have outperformed traditional approaches in feature representation and extraction and have been applied in object detection, image processing, and other computer vision assignments [42,43,44,45]. LLNet [27], a groundbreaking work for LLIE, stacked sparse denoising autoencoders for light improvement and denoising at once. Lv et al. [46] designed MBLLEN, consisting of a feature extraction, enhancement, and fusion module for facilitating the performance of LLNet. EEMEFN [47] and TBEFN [48] generated normal light pictures by fusing multiexposure images. Subsequently, the pyramid network [49,50], residual network [51], image semantic network [52], semantically contrastive learning [52], and recursive learning network [53] were introduced to enhance the feature representation and extraction of the previously reported model. Moreover, the Retinex theory and learning-based model were combined to make the proposed methods enjoy an appealing performance. For example, Retinex-Net [54] applied Enhance-Net to adjust the light of illumination maps generated by Decom-Net. A regularized sparse gradient was introduced into Retinex-Net to build a more robust LLIE approach. Wang et al. [55] applied local and global features extracted by DeepUPE to learn the mapping relationship from the original image to the illumination image. Zhang et al. [28] designed an enhancement framework (named KinD) that included three stages: a layer decomposition, reflectance recovery, and illumination light adjustment. They [56] then injected a multiscale illumination attention module into the early proposed KinD model to further promote its capacity. However, these Retinex-inspired learning methods also inevitably introduce a color deviation or hand-crafted holes due to an inaccurately estimated illumination. Additionally, the frequency-based decomposition-and-enhancement model [21] was reported to rely on the assumption that the noise exhibits different contrast at different frequency layers. Understandably, supervised methods heavily need extra computing resources to process paired (normal/abnormal) datasets for training. However, these paired images cannot be easily gathered in the real world, and we carefully capture them by artifact synthesizing or altering the exposure time and ISO rating of cameras.

Conversely, unsupervised methods are trained by unpaired images captured under various lighting conditions and scenes rather than paired images [1,29,53]. Jiang et al. [29] skillfully established EnlightenGAN [29], a typically GAN-based method, containing a global and local discriminator, self-regularized perception, and attention mechanism. Yu et al. [57] designed DeepExposure relying on reinforcement adversarial learning. However, these unsupervised methods need carefully selected unpaired images for training and inevitably introduce observable color casts. To fully explore the advantages of unsupervised and supervised methods, Yang et al. [58] presented a semisupervised approach named DRBN [59] for light enhancement. In this model, supervised learning restored the linear band representation of an enhanced image, and perceptual-quality-driven adversarial learning rearranged these linear bands to yield visually satisfying normal-light images. In [59], a network pretrained on the aesthetic dataset and an introduced LSTM module further optimized the DRBN [59]. More recently, zero-reference-based methods have proved highly efficient and cost-effective, and fewer images are needed, which has caused a stir in the fields of LLIE. For example, RRDNet [60] decomposed an image into illumination, reflectance, and noise, then the Retinex reconstruction loss, texture enhancement loss, and illumination-guided noise estimation loss were carefully contrived to drive zero-reference-based learning methods. Inspired by Retinex, Zhao et al. [30] created RetinexDIP, and Liu et al. [61] designed the RUAS network for boosting low-illumination images. Li et al. [62] employed high-order nonlinear curve mapping to adjust the image pixel values for recovering satisfying images. Afterward, they demonstrated a faster and more lightweight network called Zero DCE++ [1].

## 3. Methodology

This section first analyzes the motivation of this design. After that, the overall model framework and its main components, including frequency division (FD), the feasible feature extraction module (FFEM), and the loss function, are minutely described. We discuss the relation to other learning-based methods at the end of this section.

### 3.1. Motivation

We can easily observe images captured in insufficient light exhibit a color deviation, blurry details, and unsatisfactory brightness. Traditional LLIE methods based on HE, the Retinex theory, a fusion framework, a physical model, etc., can solve these issues to a certain extent. Still, they perform unsatisfactorily in terms of robustness. Most significantly, [17,21] showed that the detail, edge, and noise were described in the high frequencies, while the main information was demonstrated in the low frequencies. A frequency division operation can extract feature maps at different frequencies to achieve the goal of preserving detail and compressing noise. Recently, data-driven approaches based on generative adversarial networks (GANs) or convolution neural networks (CNNs) have shown strong feature representation capability, which was widely applied in image enhancement, image super-resolution, object recognition, and so on [42,43,44,45,63]. Unfortunately, although these LLIE methods significantly promote contrast, saturation, and brightness, remove the color deviation, and highlight the structural details, they heavily depend on computer resources owing to the depth or width of the network. Additionally, multiscale learning is rarely considered in these learning-based LLIE methods.

As a consequence, we combined traditional methods with CNN to design a novel LLIE method with fewer parameters and a high efficiency based on the above analysis. Specifically, we first perform frequency division on input images to achieve feature maps at high and low frequencies. Then, we propose a feasible feature extraction module containing an attention mechanism and a multiscale learning structure to improve the representation ability of our proposed CNN-based method.

### 3.2. The Overall Model Framework

**Figure 2:**
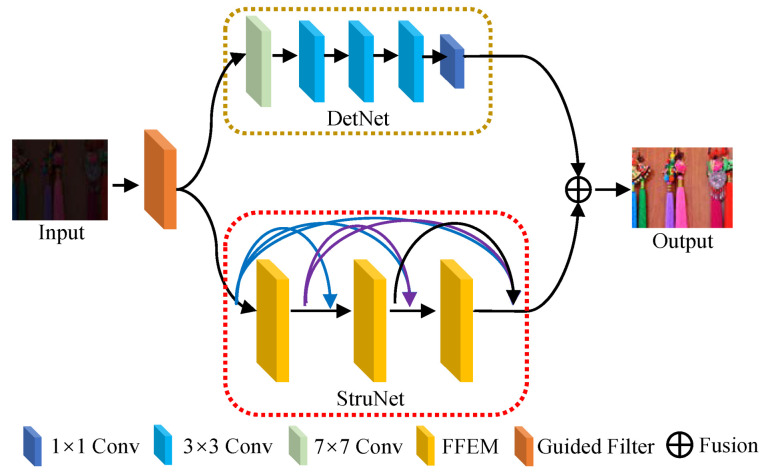
The overall framework of our presented LLIE model.

To tackle unsatisfactory contrast and brightness, blurry details, as well as the color deviation of low-light images, we present a new LLIE approach based on the theory that different information in an image is displayed at different frequencies. The overall framework of our FDMLNet, including its three main parts, i.e., frequency division (FD), DetNet, and StruNet, is illustrated in Figure 2. Among these components, FD is employed to separate the high and low frequencies of the input images; DetNet, made up of a 7×7 Conv, a 3×3 Conv, and a 1×1 Conv processes the high frequencies of the input images to preserve inherent detail and condense the noise; the low frequencies of the input images are processed by StruNet, which consists of three feasible feature extraction modules (FFEMs) to promote its brightness and contrast and remove the color deviation.

### 3.3. Frequency Division

Different frequency information plays notable roles in the whole image, and pixels with drastic changes in intensity, such as edges, detail, noise, etc., are distributed in the high frequencies, but pixels with a gentle change in intensity, such as the image structure, background, and other information, are spread over the low frequencies [21]. Based on this mechanism, this work engages a guided filter (GF) [64], an edge-preserving filter based on the local linear model, for dealing with authentic pictures to create low- and high-frequency feature maps.

Supposing that $Q_{n}$ is the $n_{th}$ input image, $I_{n}$ is the corresponding guided image, and the relationship between the output image $O_{n}$ and $I_{n}$ in the local windows $w_{k}$ tends to be linear, i.e.,

(1) $$O_{n}^{i}=a_{k}I_{n}^{i}+b_{k},\forall i\in w_{k}$$

where $w_{k}$ is a local window with a size of r×r. $a_{k}$ and $b_{k}$ are constant and their values can be calculated by minimizing the squared error between $O_{n}$ and $Q_{n}$, that is,

(2) $$E\left(a_{k},b_{k}\right)=\sum_{i\in w_{k}}\left[{\left(a_{k}I_{n}^{i}+b_{k}-Q_{n}^{i}\right)}^{2}-\varepsilon {a_{k}}^{2}\right]$$

where ε is a regularization parameter. Thus, the values of $a_{k}$ and $b_{k}$ are, respectively, defined as

(3) $$\left\{\begin{array}{cc} \; & a_{k}=\frac{\frac{1}{\left|w\right|}\sum_{i\in w_{k}}I_{n}^{i}Q_{n}^{i}-\mu_{k}{\bar{Q}}_{n,k}^{i}}{\delta_{k}+\varepsilon }\\ \; & b_{k}={\bar{Q}}_{n,k}^{i}-a_{k}\mu_{k} \end{array}\right.$$

In Equation (3), $\mu_{k}$ and $\delta_{k}$ are the pixels’ mean value and variance of the local window $w_{k}$ in the guided image, respectively. |w| is the total number of pixels in $w_{k}$. ${\bar{Q}}_{n,k}^{i}$ is the pixels’ mean value in the $n_{th}$ input image.

Since one pixel is contained in multiple windows, the average value of $a_{k}$ and $b_{k}$ is solved and Equation (1) can be rewritten as

(4) $$O_{n}={\bar{a}}_{k}I_{n}+{\bar{b}}_{k}$$

where $O_{n}$ is the low-frequency feature map of the input image. Therefore, its high-frequency feature map $P_{n}$ is

(5) $$P_{n}=Q_{n}-O_{n}$$

### 3.4. Feasible Feature Extraction Module

**Figure 3:**
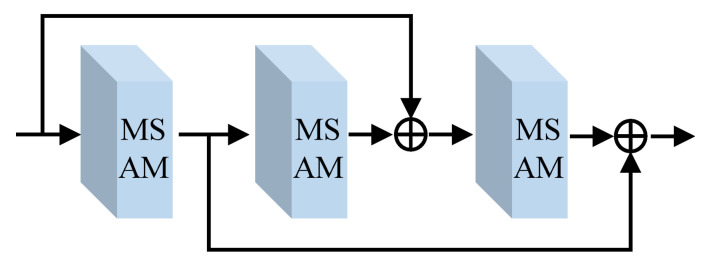
The structure of a feasible feature extraction module (FFEM).

Nowadays, we have a detailed analysis of the feasible feature extraction module (FFEM) structure, which is depicted in Figure 3. This module stacks 3 MSAMs in an updated dense skip-connection way to promote the learning ability of FEMLNet and fully explore features at different levels. The process can be expressed as

(6) $$O_{n}^{d}=O{}_{n,m-1}^{d}+O_{n,m+1}^{d}\left(O_{n}^{d-1}+O_{n,m}^{d}\right)$$

where $O_{n}^{d}$ and $O_{n}^{d-1}$ are the $n_{th}$ output images of the $d_{th}$ and ${d-1}_{th}$ FFEM, respectively. $O_{n,m-1}^{d}$, $O_{n,m}^{d}$, and $O_{n,m+1}^{d}$ are, respectively, the output results of the ${m-1}_{th}$, $m_{th}$, and ${m+1}_{th}$ MSAM in the $d_{th}$ FFEM.

**Figure 4:**
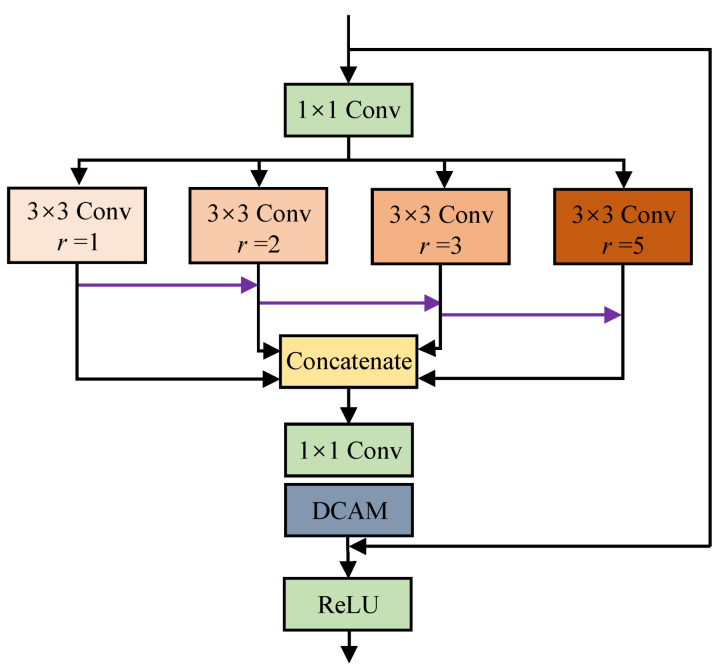
The structure of the multiscale learning block with dual-branch channel attention (MSAM).

Multiscale learning structure: Generally, the image exhibits different characteristics at various scales, and a multiscale representation can effectively extract its information at different scales and promote the performance of learning-based methods [15,56]. As a result, the multiscale learning strategy has broadly been conducted on object identification, pose recognition, face detection, and other computer vision tasks [42,43,44,45]. However, this strategy is rarely considered in most state-of-the-art LLIE models. In this proposed FDMLNet, we built an efficient multiscale learning structure called MSAM, which consists of a multiscale learning block and a dual-branch channel attention mechanism. This MSAM consists of small convolution kernel groups with a size of 3×3 and different dilation rates, i.e., 1, 2, 3, and 5. Furthermore, Figure 4 demonstrates its structure in detail.

The image dimensionality is reduced by the 1×1 convolution operation to alleviate the computational load. Then, we extract multiscale information through four parallel branches, which are made up of 3×3 convolutions with dilation rates r=1,2,3, and 5, respectively. Notably, the features extracted by the previous branch are injected into the next branch to adequately utilize the image’s potentially multiscale information. The extraction procedure of the multiscale feature can be described as

(7) $${Fu}_{out}=Con_{3}\left(f_{1}\right)+Con_{3}\left(f_{2}\right)+\cdots +Con_{3}\left(f_{i}\right),i\leq 4$$

In the following, we integrate the results of the four branches by concatenating them and then, a 1×1 convolution operation is used to process the concatenated results. Finally, the dual-branch channel attention mechanism processes the convolution results, and then the output features are injected into input images to exploit more inherent global and local information.

**Figure 5:**
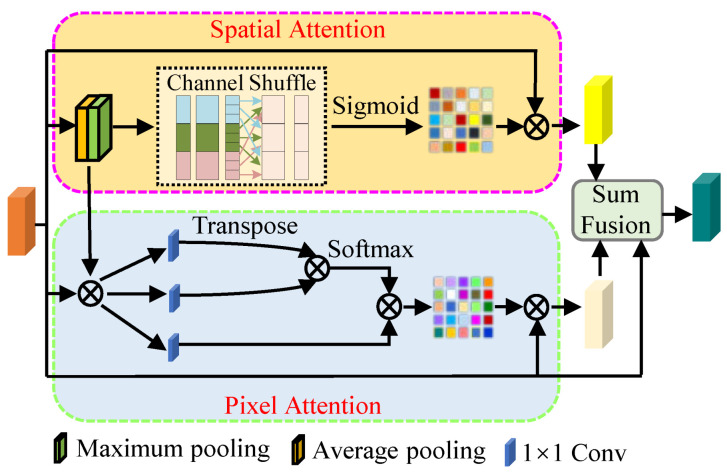
The structure of the dual-branch channel attention mechanism (DCAM).

Dual-branch channel attention mechanism: As we all know, the human brain selectively focuses on the key information while ignoring the rest of the visible information [1,7,21,29,43]. The attention mechanism, a strategy mimicking the human brain, has been widely used for generating attention-aware features and extracting key information for promoting the ability of CNN-based methods by adaptively rearranging weights. We designed a dual-branch channel attention mechanism, containing pixel and spatial attention mechanisms, to further enhance the performance of this proposed FDMLNet, and Figure 5 shows its structure in detail. We can observe this design can fully exploit the image features in different channels.

Specifically, we send the input data into a spatial attention branch to extract both the background and texture of the image. Firstly, average pooling and max pooling operations are used to process the input data, and then we fuse them in an additive manner. Suppose that the size of the input data is H×W, the united feature map $z_{c}$ is defined as

(8) $$\begin{array}{cc} z_{c} & =H_{avgp}+H_{maxp}\\ \; & =\frac{\sum_{i=1}^{H}\sum_{j=1}^{W}u_{c}\left(i,j\right)}{H\times W}+\max_{i,j\in H\times W}u_{c}\left(i,j\right) \end{array}$$

where $H_{avgp}$ and $H_{maxp}$ are average pooling and max pooling operations, respectively. $u_{c}\left(i,j\right)$ is the pixel value at position (i,j) in the input data.

Then, the 7×7 Conv with an activation function (sigmoid) is used to calculate the spatial weight map $W_{s}$, i.e.,

(9) $$W_{s}=sig\left(Conv_{7\times 7}\left(z_{c}\right)\right)$$

where $Conv_{7\times 7}$ is a convolution with a size of 7×7, sig is the sigmoid function, an activation function, and a channel shuffle is introduced to tackle the communication of feature maps among different groups. Then, we extract the image’s spatial feature $F_{s}$ by multiplying the input data with the weight map, namely $F_{s}=u_{c}\times W_{s}$.

In the pixel attention branch, the feature map $z_{c}$ that fuses features generated by the average pooling and max pooling operations is added into the input data $u_{c}$ to avoid the influence of the spatial relationship and is recorded as $v_{c}$. Then, three 1×1 Conv operations are applied to $v_{c}$ and the result of the top branch is processed by a transpose operation. In order to solve the weighted matrices ${W^{\prime }}_{p}$, the transposed result was multiplied by the result of the second branch and then processed by a softmax function. The above procedure can be described as,

(10) $${W^{\prime }}_{p}=soft\left({\left(Conv_{1\times 1}\left(v_{c}\right)\right)}^{T}\times \left(Conv_{1\times 1}\left(v_{c}\right)\right)\right)$$

where soft is the softmax function and $Conv_{1\times 1}$ is the convolution with the size of 1×1.

Subsequently, the result of the final branch is multiplied by the weighted matrices ${W^{\prime }}_{p}$ to calculate the pixel weighted map $W_{p}$,

(11) $$W_{p}={W^{\prime }}_{p}\times Conv_{1\times 1}\left(v_{c}\right)$$

The pixel weighted map $W_{p}$ and the spatial weight map $W_{s}$ are integrated in a sum operation to obtain attention-aware feature maps. Furthermore, the input data are fused with the attention-aware feature maps to entirely explore its inherent information *F*, that is

(12) $$F=sum\left(u_{s},sum\left(W_{p},W_{s}\right)\right)$$

### 3.5. Loss Function

To guarantee our method shows satisfactory performance in LLIE, we carefully devised a hybrid loss function containing a structure similarity (SSIM) loss, $L_{1}$ loss, total variation (TV) loss, and color constancy (CC) loss to assess the discrepancy between the output and authentic images. These four loss functions are minutely described as follows:

$L_{1}$-norm loss: We first calculate the mean absolute error (i.e., $l_{1}$-norm) between the output result $I_{out}$ and normal-light image $I_{nl}$ to measure their difference. It can be calculated as follows:

(13) $$L_{l_{1}}={∥I_{out}-I_{nl}∥}_{1}$$

Structure similarity (SSIM) loss: The $L_{1}$-norm loss can make our model generate high-illumination images, but over- or underenhancement and other structural distortion are introduced in the enhanced images. To address these challenging issues, we injected the SSIM loss to examine the structure similarity. The formula of the SSIM loss is shown below:

(14) $$L_{SSIM}=1-\frac{2\mu_{x}\mu_{y}+c_{1}}{\mu_{x}^{2}+\mu_{y}^{2}+c_{1}}\cdot \frac{2\sigma_{xy}+c_{2}}{\sigma_{x}^{2}+\sigma_{y}^{2}+c_{2}}$$

where $\mu_{x}$ and $\mu_{y}$ are the mean values of the pixels in the output and input images, respectively. $\sigma_{x}$ and $\sigma_{y}$ stand for the pixels’ variance of the output and input images, respectively. $c_{2}$ and $c_{2}$ are constants, which were empirically set as 0.0001 and 0.0009.

Total variation (TV) loss: Although most data-driven approaches effectively light up low-illumination images, they inevitably generate observable noise. For compressing the image noise, the TV loss was applied to smooth the output image by minimizing its gradient in our method, and its definition is:

(15) $$L_{TV}=\sum_{i=1}^{H}\sum_{j=1}^{W}\sqrt{\left(P_{i,j}-P_{i+1,j}\right)\left(P_{i,j}-P_{i,j+1}\right)}$$

where *H* and *W* are the image size. *P* is a pixel value. *i* and *j* are the pixel indexes in the enhanced image.

Color constancy (CC) loss: Generally speaking, low-light images encounter a color deviation, which leads to an unsatisfactory visual appearance. This work introduced the CC loss function proposed in [62] to fully explore the relationship among R, G, and B channels and correct the distorted color. The CC loss function can be defined as

(16) $$L_{CC}=\sum_{\forall \left(p,q\right)\in \varepsilon }{\left(J_{p}-J_{q}\right)}^{2},\varepsilon \in \left\{\left(R,G\right),\left(R,B\right),\left(G,B\right)\right\}$$

where $J_{\cdot }$ is the mean value of the *p* or *q* channel in the output result. $\left(p,q\right)$ stands for a pair of channels.

Total loss: We integrated the above-listed four loss functions to design a total loss function, named $L_{total}$, defined as:

(17) $$L_{Total}=L_{l_{1}}+L_{SSIM}+\omega_{TV}L_{TV}+\omega_{CC}L_{CC}$$

where $L_{l_{1}}$, $L_{SSIM}$, $L_{TV}$, and $L_{CC}$ are the $l_{1}$-norm, SSIM, TV, and CC losses, respectively. $\omega_{TV}$ and $\omega_{CC}$ are the weights, set as 0.8 and 0.4.

### 3.6. Relation to Other Learning-Based Methods

Relation to Xu et al. [21]: The proposed method relied on the same mechanism (i.e., the image exhibits different features at various frequency layers) as the literature [21]. However, the description of three apparent differences between these two methods is as follows:

(1) The way the frequency division was performed: Xu et al. [21] employed a learning-based way, paying attention to the context encoding model (ACE), to adaptively decompose the high and low frequencies of the input image. However, a guided filter, a traditional preserving filer, was applied to achieve the image’s high and low frequencies in our work.
(2) The way the enhancement was performed: Xu et al. [21] compressed the inherent noise and highlighted the details by the cross-domain transformation (CDT) model. However, we designed two subnets, i.e., DetNet and StruNet, to enhance the image, and the former processed the high-frequency components of the image to highlight its detail while the latter disposed of its low-frequency components to generate visually pleasing structural images.
(3) Furthermore, we injected spatial attention and pixel attention mechanisms into our reported FDMLNet to fully exploit the inherent information in the image. In addition, the multiscale structure was also embedded to promote the multiscale representation ability of the proposed model.

Relation to PRIEN [50]: PRIEN [50] employed a dual-attention mechanism to promote its performance in LLIE. In this paper, we created a dual-branch channel attention module integrating spatial and pixel relationships. Noticeably, a channel shuffle was introduced in the spatial attention branch to achieve communication among all channels, and the pixels’ spatial relationship of the image was injected into the pixels’ attention branch. In addition, [50] only considered the SSIM loss function, which may magnify the inherent noise or distort the image color. However, the SSIM loss function, TV loss, L1 loss, and color loss functions were all brought into our model to remove the color deviation, preserve the details, and compress the inherent noise.

## 4. Experimental Results and Analysis

In this part, we describe the experimental results and analysis in detail. Firstly, we briefly present the implementation details and experimental settings. Then, ablation studies, as well as qualitative and quantitative assessments on paired and unpaired datasets, are depicted. To this end, the analysis of the application test is implemented.

### 4.1. Experimental Settings

In the following, we state the comparison approaches, public benchmarks, and assessment criteria in detail.

Comparison approaches: We carefully selected 12 state-of-the-art approaches as comparison methods for validating the superiority of this FDMLNet for light enhancement. These selected methods contained three traditional methods, i.e., LR3M [18], simultaneous reflection and illumination estimation (SRIE) [19], and the bioinspired multiexposure fusion framework (BIMEF) [20]; seven supervised-learning-based methods, i.e., RetinexNet [54], deep stacked Laplacian restorer (DSLR) [49], KinD [28], DLN [14], DRBN [59], SCL-LLE [52], and MIRNet [65]; an unsupervised-learning-based method, i.e., EnlightenGAN [29]; and a zero-reference-learning-based method, i.e., Zero DCE++ [1]. Notably, three traditional methods were coded in Matlab and the other eight comparison methods were coded in Python and Pytorch.

Public benchmarks: We performed verification experiments on two paired datasets (LOL and MIT-Adobe FiveK) and four unpaired datasets (LIME, MEF, NPE, and VV) to test their performance in light enhancement. The LOL dataset was captured by changing the exposure time and ISO of a camera and contains 500 pairs of abnormal/normal light RGB-images with a size of 400 × 600. The MIT-Adobe FiveK benchmark contains 5000 RAW-images processed by five professional photographers. Adobe Lightroom was used to transform these images from the RAW to the RGB format to train the LLIE models. The LIME, MEF, NPE, and VV benchmarks contain 10, 17, 84, and 24 images, respectively.

Assessment criteria: We adopted four full-reference commonly used criteria, including the mean square error (MSE), peak signal-to-noise ratio (PSNR), structural similarity index measure (SSIM) [66], and learned perceptual image patch similarity (LPIPS) [67] to assess these LLIE comparison methods on the LOL and MIT-Adobe FiveK datasets. For these criteria, an MSE, PSNR, or LPIPS [67] value, as well as a higher PSNR value indicated a better visual perception. Furthermore, two nonreference criteria, i.e., the natural image quality evaluator (NIQE) [13] and patch-based contrast quality index (PCQI), were employed to assess the performance of these LLIE methods on the LIME, MEF, NPE, and VV public benchmarks, and a lower NIQE [13] or higher PCQI score suggested more satisfying enhanced images.

### 4.2. Training Details

We carried out our designed model on a platform with two 2080Ti GPUs, a Windows 10 operating system, 128 GB of RAM, and an Intel(R) Core(TM) i7-9700K CPU @ 3.60 GHz. This proposed network was coded in Pytorch and optimized by stochastic gradient descent (SGD). Furthermore, the batch size was 8, the learning rate was 0.0001, and the activate function was ReLU. We randomly selected 485 paired images from the LOL dataset for training our model. Finally, the MIT-Adobe, LOL test, LIME, MEF, NPE, and VV benchmarks were also selected for the testing experiment.

### 4.3. Ablation Studies

**Figure 6:**
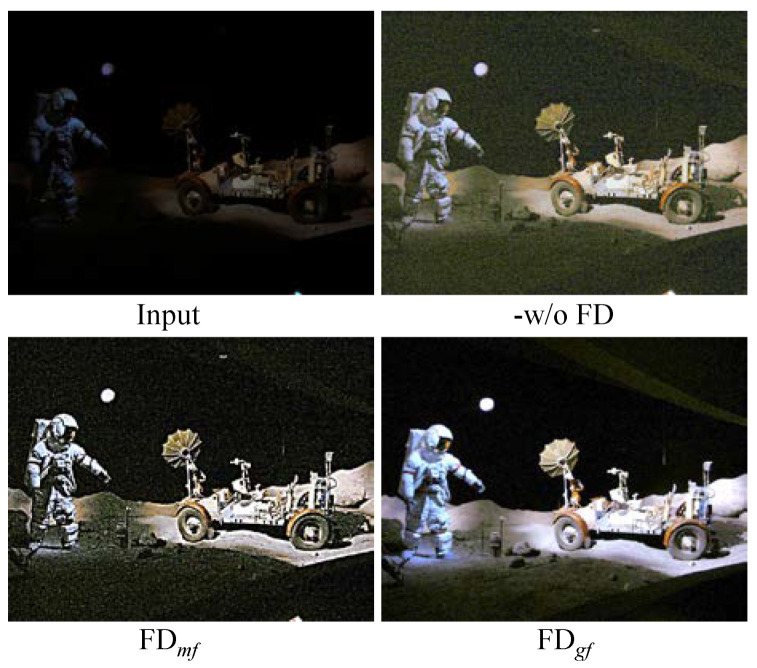
Visual comparison of frequency division by different means.

Ablation studies on the frequency division, multiscale learning, dual-branch channel attention mechanism, loss and activation functions were conducted to fully understand the FDMLNet. These ablation studies are detailed as follows:

Study of the frequency division: Figure 6 describes the visual enhancement results to verify the effectiveness of the frequency division (FD) operation in our presented FDMLNet model. Among them, -w/o FD represents our designed model without FD operation, FD${}_{mf}$ and FD${}_{gf}$ stand for our developed model employing a mean filter (mf) and a guided filter (gf) to separate the image high and low frequencies, respectively. From the results, we discover that FD could avoid color casts and FD${}_{mf}$ inevitably introduced observable noise. However, FD${}_{gf}$ coinstantaneously compressed the inherent noise and lights up the image.

**Figure 7:**
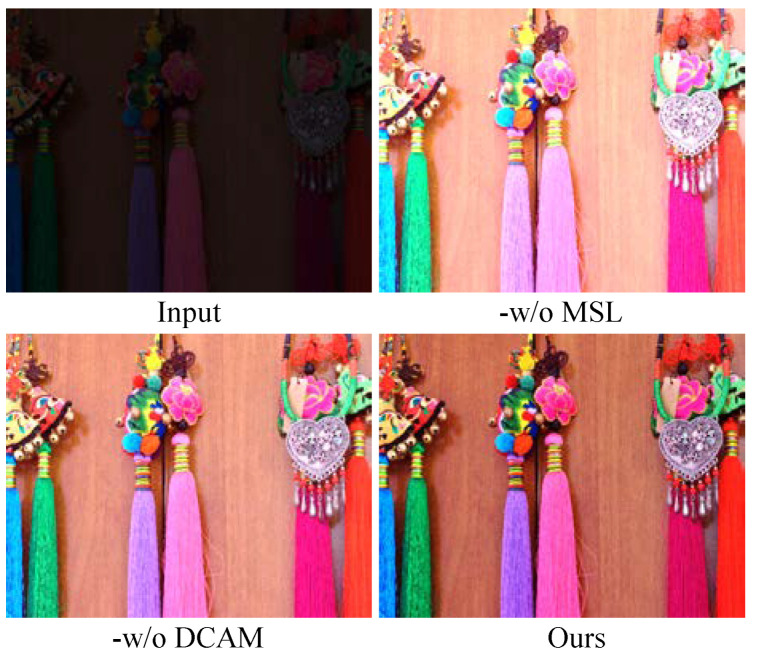
Qualitative analysis of every components in our model.

Study of the multiscale learning structure: To examine the multiscale learning (MSL) structure of our method, MSL was removed (named -w/o MSL). That is to say, our model only extracted the image information under a single scale. Notice that -w/o MSL yielded unwanted light and color casts in the enhanced images, as shown in Figure 7. Additionally, from Table 1, we see FDMLNet generated higher PSNR and SSIM scores on both the LOL and MIT-Adobe FiveK benchmarks. Thus, MSL improved absolutely the ability of our model in LLIE.

Study of the dual-branch channel attention mechanism: -w/o DCAM indicates that the attention mechanism was not taken into account in our model. As depicted in Figure 7, -w/o DCAM failed to enhance local details and remove the color deviation as well as hand-crafted halos. However, the output image generated by our method showed a high brightness, vivid colors, and clearer details. The PSNR and SSIM [66] of the different operations on the LOL and MIT-Adobe datasets are shown in Table 1; it can be seen that our method generated the highest scores of two elevation criteria on the selected public datasets.

**Figure 8:**
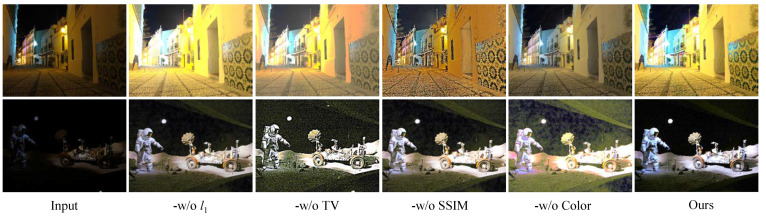
Visual comparison of the loss function in the presented FDMLNet approach.

Study of the loss function: We studied the roles of the mentioned loss functions in our design. Furthermore, -w/o L1, -w/o TV, -w/o SSIM, and -w/o CC indicates that the L1 loss, TV loss, SSIM loss, and CC loss were removed in our loss function, respectively. Figure 8 demonstrates the image improved by our model with different loss functions, and Table 2 shows the PSNR and SSIM [66] scores of two public benchmarks processed by our FDMLNet model with different operations. Compared with other operations, we easily find that only our design exhibited the best performance in both quantitative and qualitative analyses for light enhancement.

**Figure 9:**
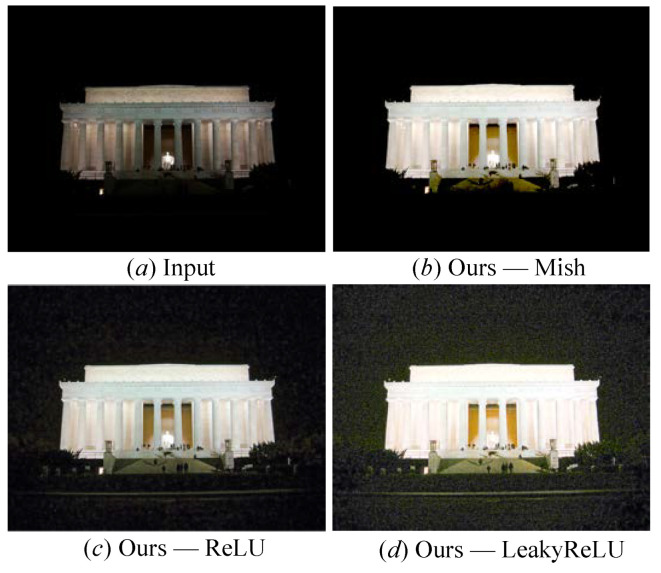
The image processed by our method with different activation functions.

Study of the activation function: To study the performance of the presented FDMLNet with different activation functions, we show the processed images by our method with LeakyReLU, Mish, and ReLU in Figure 9. We find that LeakyReLU amplified the dark area’s inherent noise, and Mish was unsatisfactory for enhancing the local dark area. However, ReLU could compress the image noise and light up the whole image simultaneously. Furthermore, it was so intuitive and so sensible that both LOL and MIT-Adobe FiveK datasets enhanced by FDMLNet showed optimal PSNR and SSIM values [66], as seen from Table 2.

### 4.4. Comprehensive Assessment on Paired Datasets

**Figure 10:**
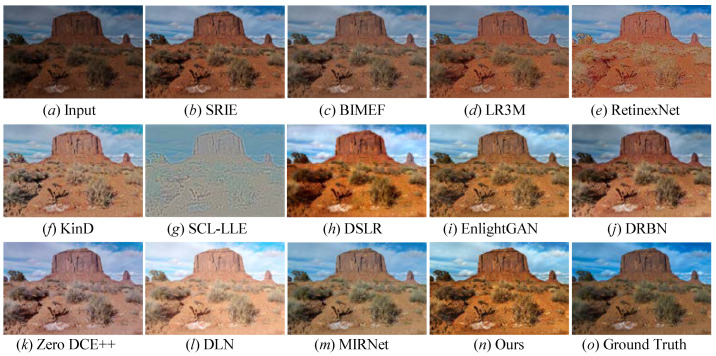
Visual comparisons of different approaches on the MIT-Adobe benchmark. (**a**) Low-light image selected from MIT-Adobe benchmark, (**o**) the corresponding ground truth image. The enhancement result via (**b**) SRIE [19], (**c**) BIMEF [20], (**d**) LR3M [18], (**e**) RetinexNet [54], (**f**) KinD [28], (**g**) SCL-LLE [52], (**h**) DSLR [49], (**i**) EnlightenGAN [29], (**j**) DRBN [59], (**k**) Zero DCE++ [1], (**l**) DLN [14], (**m**) MIRNet [52], and (**n**) Ours.

Qualitative evaluation: We first applied the FDMLNet and comparison LLIE methods on the MIT-Adobe 5K and LOL paired benchmarks to validate their effectiveness in terms of light enhancement. The qualitative evaluation on these two datasets was as follows:

Figure 10 shows the enhanced images of every comparison LLIE methods on the image randomly selected from the MIT-Adobe paired benchmark. The following observations could be obtained: First, the LLIE methods succeeded in lighting up low-illumination images, indicating that the image enhancement was an effective way to tackle the issues of these images. However, SRIE [19], BIMEF [20], and LR3M [18] could not generate the wanted images with a satisfactory visual appearance. RetinexNet [54] improved the illumination of images while yielding unnatural visual experiences. KinD [28] failed to recover the inherent details and introduced unsatisfactory color casts in local dark regions of the image. SCL-LLE [52] generated undesired images with an unnatural visual experience (observed in picture *g* in Figure 10). MIRNet [52] succeeded in improving the image brightness, but the enhanced images exhibited a color deviation and low contrast. DSLR-enhanced images had a blocking effect, and DRBN-enhanced pictures encountered color distortion (discovered in the sky part of the images *h* and *j* in Figure 10). EnlightGAN [29] failed to remove the artifacts’ halos and blocking effects. We also found that DLN [14] was unsatisfactory in removing whitish tone and correcting color distortion. Although Zero DCE++ [1] could successfully light up the image, it brought in unnatural visual and blurry details. Compared with twelve state-of-the-art LLIE methods, only our method showed an impressive performance in rebuilding artifact-free images with a visually pleasing appearance, clearer details, and vivid colors.

**Figure 11:**
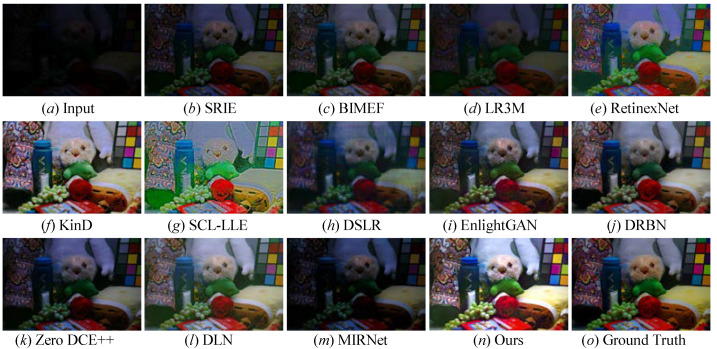
Visual comparisons of different approaches on the LOL benchmark. (**a**) lowlight image selected from LOL benchmark, (**o**) the corresponding ground truth image. The enhancement result via (**b**) SRIE [19], (**c**) BIMEF [20], (**d**) LR3M [18], (**e**) RetinexNet [54], (**f**) KinD [28], (**g**) SCL-LLE [52], (**h**) DSLR [49], (**i**) EnlightenGAN [29], (**j**) DRBN [59], (**k**) Zero DCE++ [1], (**l**) DLN [14], (**m**) MIRNet [52], and (**n**) Ours.

All the mentioned LLIE methods were also compared on the LOL public benchmark, and a randomly selected result from the LOL dataset is shown in Figure 11. From Figure 11, we easily discover that these comparison methods either failed to light up local darkness or introduced unwanted visual appearances, such as hand-crafted halos, blocking effects, and so on. Specifically, DSLR [49], DRBN [59], and EnlightenGAN [29] inevitably distorted the color of some low-light photos; RetinexNet [54] generated unwanted artifacts holes; SRIE [19], BIMEF [20], and LR3M [18] could not effectively light up the low-illumination image; KinD [28] amplified the inherent noise; SCL-LLE [52] generated high-light images, while their appearance was not natural. MIRNet [65] showed a poor performance in lighting up the illumination of some low-light images (as seen in picture *m* in Figure 11). Here, our proposed method not only could effectively light up the low-illumination image, but could also eliminate distorted colors as well as promote the image quality with clearer details and a corrected exposure. That is, our method outperformed all the mentioned comparison methods in LLIE.

Quantitative evaluation: In addition to the visual comparison listed above, a quantitative evaluation was also performed on the LOL and MIT-Adobe public benchmarks to further validate our designed model comprehensively. The average MSE, SSIM [66], PSNR, and LPIPS [67] scores on these two public datasets promoted by the aforementioned LLIE models are shown in Table 3. For the four reference criteria, we can readily easily notice that SRIE [19], BIMEF [20], and LR3M [18] were inferior to some data-driven approaches, which empirically indicated that the latter showed an impressive performance in LLIE owing to its strong ability for feature representation and extraction. In comparison, among all the aforementioned methods, our FDMLNet method generated comparable scores of MSE, SSIM [66], PSNR, and LPIPS [67] in these two datasets. This means our proposed method performed well in lighting up the brightness, preserving inherent details, and compressing the noise of low-light images in terms of both quantitative and qualitative evaluations.

### 4.5. Comprehensive Assessment on Unpaired Datasets

**Figure 12:**
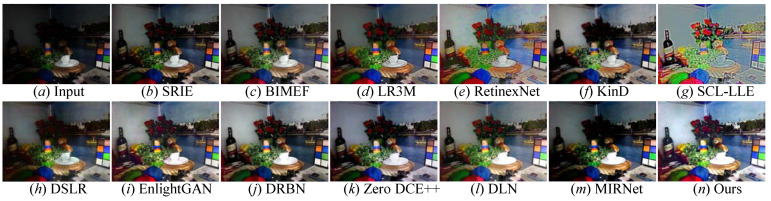
Visual comparisons of different approaches on the LIME benchmark. (**a**) Low-light image selected from LIME benchmark. The enhancement result via (**b**) SRIE [19], (**c**) BIMEF [20], (**d**) LR3M [18], (**e**) RetinexNet [54], (**f**) KinD [28], (**g**) SCL-LLE [52], (**h**) DSLR [49], (**i**) EnlightenGAN [29], (**j**) DRBN [59], (**k**) Zero DCE++ [1], (**l**) DLN [14], (**m**) MIRNet [52], and (**n**) Ours.

**Figure 13:**
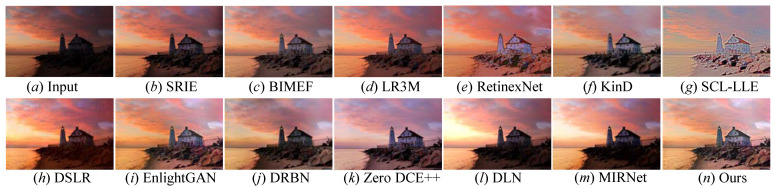
Visual comparisons of different approaches on the MEF benchmark. (**a**) Low-light image selected from MEF benchmark. The enhancement result via (**b**) SRIE [19], (**c**) BIMEF [20], (**d**) LR3M [18], (**e**) RetinexNet [54], (**f**) KinD [28], (**g**) SCL-LLE [52], (**h**) DSLR [49], (**i**) EnlightenGAN [29], (**j**) DRBN [59], (**k**) Zero DCE++ [1], (**l**) DLN [14], (**m**) MIRNet [52], and (**n**) ours.

**Figure 14:**
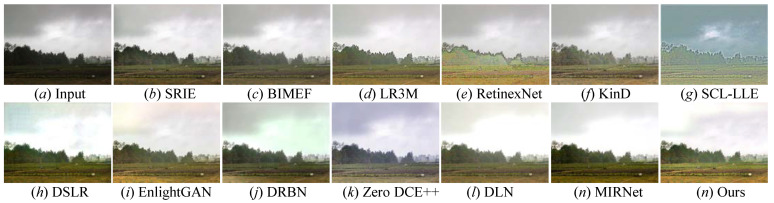
Visual comparisons of different approaches on the NPE benchmark. (**a**) Low-light image selected from NPE benchmark. The enhancement result via (**b**) SRIE [19], (**c**) BIMEF [20], (**d**) LR3M [18], (**e**) RetinexNet [54], (**f**) KinD [28], (**g**) SCL-LLE [52], (**h**) DSLR [49], (**i**) EnlightenGAN [29], (**j**) DRBN [59], (**k**) Zero DCE++ [1], (**l**) DLN [14], (**m**) MIRNet [52], and (**n**) ours.

**Figure 15:**
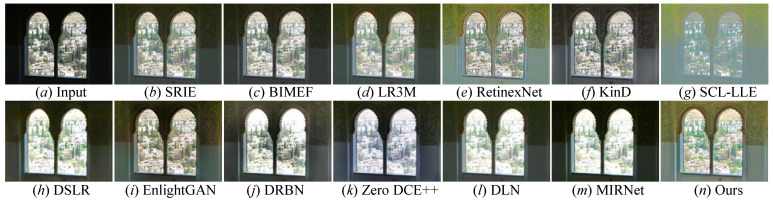
Visual comparisons of different approaches on the VV benchmark. (**a**) Low-light image selected from VV benchmark. The enhancement result via (**b**) SRIE [19], (**c**) BIMEF [20], (**d**) LR3M [18], (**e**) RetinexNet [54], (**f**) KinD [28], (**g**) SCL-LLE [52], (**h**) DSLR [49], (**i**) EnlightenGAN [29], (**j**) DRBN [59], (**k**) Zero DCE++ [1], (**l**) DLN [14], (**m**) MIRNet [52], and (**n**) ours.

Qualitative evaluation: To effectively and comprehensively examine the light enhancement capability of state-of-the-art comparison methods and our FDMLNet, four unpaired benchmarks (i.e., LIME, MEF, NPE, and VV) were also used to conduct validation experiments. We demonstrate randomly selected results generated by these cutting-edge approaches from the LIME, MEF, NPE, and VV benchmarks in Figure 12, Figure 13, Figure 14 and Figure 15, respectively. From these enhanced images, the following observations can be made: BIMEF [20], a fusion-strategy-based method, tried to produce high-light images by fusing multiexposure images. Significantly, this method failed to light up the dark regions of some pictures and introduced observable over- or underenhancements. Both LR3M [18] and SRIE [19] could notably promote the image brightness and contrast, but LR3M-enhanced images suffered from unsatisfactory structural details and SRIE [19] excessively enhanced some images causing local overexposure. RetinexNet [54] introduced unsatisfactory artifact holes, DSLR [49] generated an unnatural visual appearance, blocking effects, and color casts. Zero DCE++ [1] and DLN [14] effectively enhanced low-illumination images with blurry details and low contrast, but they all introduced an additional whitish tone in the enhanced images. Additionally, the former generated unwanted hand-crafted holes and blurry edges in some enhanced images, and the latter was not satisfactory when tackling color distortion. SCL-LLE [52] generated visually unnatural images, and MIRNet [65] failed to address the local darkness of the enhanced images. Although EnlightenGAN [29] and DRBN [59] were satisfactory for lighting up the brightness of low-light images, they inevitably brought in some local underenhancement or darkness and unsatisfactory edges. On the contrary, our discovered method showed a satisfactory manifestation in lighting illumination, preserving edges and structural details, avoiding color distortion, and over- or underenhancement on the LIME, MEF, NPE, and VV unpaired benchmarks. To wit, our method outperformed all aforementioned comparison approaches in lighting up low-light images.

Quantitative Evaluation: For the LIME, MEF, NPE, and VV unpaired benchmarks, we first conducted a visual comparison and analysis on images generated by different methods in the previous section. Subsequently, the NIQE [13] and PCQI nonreference assessment metrics were applied to objectively assess the enhanced images in terms of a quantitative evaluation. Furthermore, we show the average quantitative (NIQE [13], and PCQI) scores for state-of-the-art comparison methods on the LIME, MEF, NPE, and VV datasets in Table 4. We can conclude the following: These datasets enhanced by SRIE [19], BIMEF [20], and LR3M [18] exhibited lower values on all aforementioned nonreference criteria, indicating that conventional methods performed unsatisfactory in LLIE. Conversely, our designed FDMLNet generated higher scores of the NIQE [13] and lower scores of the PCQI assessment criteria in the LIME, MEF, NPE, and VV datasets than other state-of-the-art comparison approaches. In a nutshell, the proposed FDMLNet model generally performed satisfactorily in contrast stretch, color correction, and detail preservation for addressing the challenging issues of low-illumination pictures.

### 4.6. Comprehensive Analysis of Computational Complexity

We show the computational complexity of all above-listed methods and their average execution time on the LOL benchmark in Table 5. From the table, we find Zero DCE++ [1] enjoyed the fewest number of parameters and flops, the fastest speed owing to its estimating of the parameters of the high-order curve via a lightweight network. Besides Zero DCE++ [1], DRBN [59], and RetinexNet [54], our FDMLNet exhibited a fewer number of parameters and faster speed in light enhancement than the remaining comparison approaches. However, all the validation experiments proved that our FDMLNet outperformed all comparison methods in LLIE.

### 4.7. Comprehensive Assessment on Real Images

**Figure 16:**
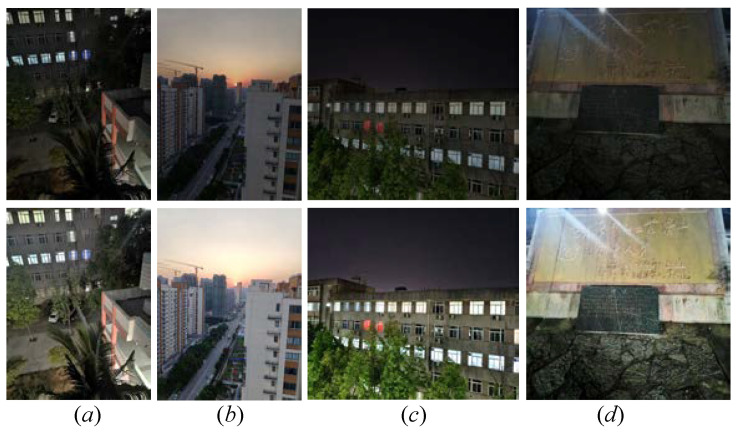
Visual comparisons of the FDMLNet tested on real low-light images. (**a**,**b**) captured by Mate 20 Pro, (**c**,**d**) captured by Vivo X60. From top to bottom, the first row is the original image, and the second row is the result generated by our method.

**Figure 17:**
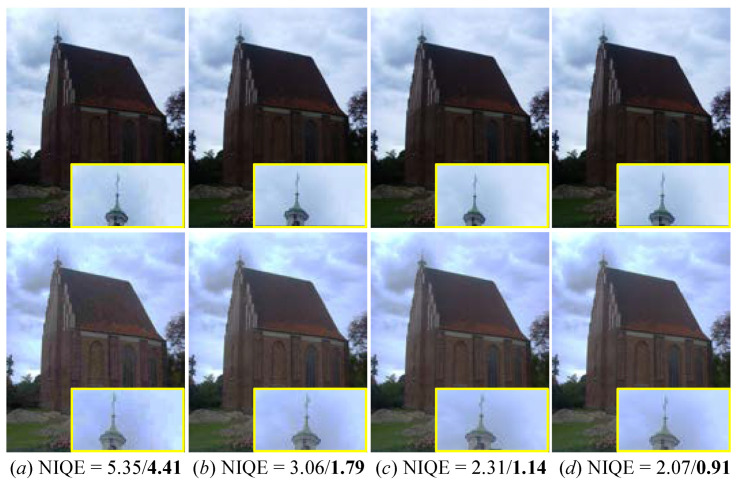
Visual comparisons of the FDMLNet tested on compressed low-light images. (−/− is the NIQE score of the original/enhanced image). From left to right, the compression ratios are set to 0.2, 0.5, 0.8 and 1, respectively. From top to bottom, the first row is the original image, and the second row is the result generated by our method.

To prove the application of our method in real-world images, we applied our FDMLNet on real low-light images captured by Mate 20 Pro and Vivo X60 phones. The results yielded by our the FDMLNet are depicted in Figure 16. The following observation can be obtained: the enhanced images consistently exhibited a visually pleasing appearance, vivid colors, and more apparent details with the help of our designed learning-based method. Therefore, our proposed FDMLNet model could be applied to promote the quality of images received from a common phone camera, such as a Mate 20 Pro, Vivo X60, and so on. Additionally, we processed the compressed low-light images, which were created by setting the compression ratios to 0.2, 0.5, 0.8, and 1 in order to test our method. The enhanced images and the NIQE (original/enhanced images) are shown in Figure 17. We can easily find that the proposed FDMLNet generated more satisfactory images and had lower NIQE scores under a variety of compression ratios. Unfortunately, our proposed method failed to remove the hand-crafted halos, especially with a compression ratio of 0.2 (observed in picture *a* in Figure 17).

## 5. Discussion and Limitation

**Figure 18:**
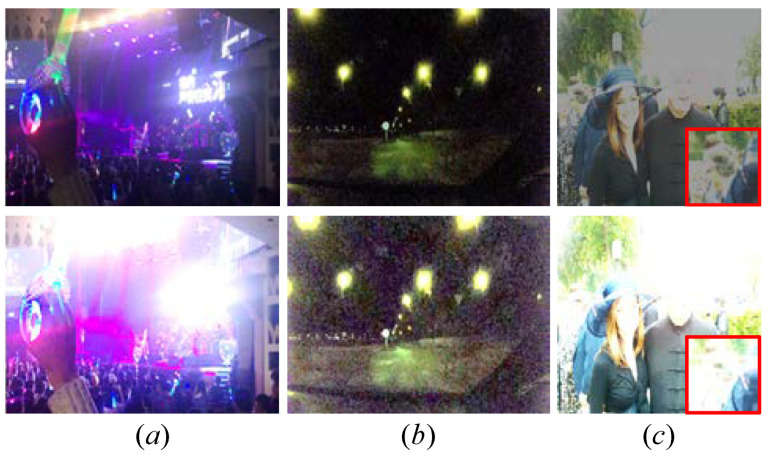
Visual comparisons of the FDMLNet tested on different low-light images. Low-light image with (**a**) colored light, (**b**) boosted noise, (**c**) local overexposure. From top to bottom, the first row is the original image, and the second row is the result generated by our method.

Low-illumination images not only exhibit an unsatisfactory visual appearance but deliver compromised information for other high-level computer vision applications. Hence, it is urgent but practical to improve their quality. Our FDMLNet required fewer parameters, had a faster speed, and performed well in generating a visually pleasing image in most cases, but it still showed some limitations in certain unique scenes. For example, Figure 18 demonstrates the visual comparisons of the FDMLNet tested on different low-light images; we can observe that our method failed to restore the quality of the images with excessive noise, colored light, and local overexposure. The most probable reason was that our designed DetNet was without a denoising operation and directly processed the image’s high frequencies containing inherent noise. Moreover, some special scene images, such as colored light images, were not included when training our model. In the future, we will tackle these challenging issues by fusing semantic information and building a diversity dataset to train the model.

## 6. Conclusions

We constructively demonstrated a novel and highly efficient method for tackling the challenging issues of low-illumination photos. This proposed FDMLNet first employed a guided filter to separate the image high and low frequencies. In addition, the DetNet and StruNet were separately used to process them for enhancing low-light images. In StruNet, a multiscale learning block with a dual-branch channel attention strategy was injected to fully exploit the information at different scales. Then, the FFEM was composed by three MSAMs in a improved skip-connection way to utilize the hierarchical and inherent features. Furthermore, the FFEMs were connected by means of a dense connection to guarantee the multilevel information was completely assimilated. Extensive experimental validation results on several public paired/unpaired benchmarks proved that our FDMLNet was superior to state-of-the-art approaches in terms of LLIE. However, our method ineffectively recovered the color and brightness of images with boosted noise or colored light; we will tackle these remaining problems in the future.

## Figures and Tables

**Table 1:** The PSNR and SSIM [66] of different operations on LOL and MIT-Adobe datasets. Bold text means the best performance.

Method	LOL		MIT-Adobe	
	PSNR ↑	SSIM ↑	PSNR ↑	SSIM ↑
-w/o MSL	24.519	0.875	17.845	0.881
-w/o DCAM	24.545	0.880	17.887	0.886
Ours	**24.658**	**0.886**	**17.895**	**0.879**

**Table 2:** The PSNR and SSIM [66] of our method under different loss and activation functions on LOL and MIT-Adobe datasets. Bold text means the best performance. ↑ represents the bigger the value, the better the performance.

	Function	LOL		MIT-Adobe	
		PSNR ↑	SSIM ↑	PSNR ↑	SSIM ↑
Loss	-w/o L1	24.644	0.880	21.359	0.866
	-w/o TV	24.599	0.879	21.353	0.869
	-w/o SSIM	23.979	0.869	21.288	0.858
	-w/o Color	24.656	0.881	21.358	0.870
	Our	**24.658**	**0.886**	**21.361**	**0.879**
Activate	LeakyReLU	24.317	0.877	21.105	0.867
	Mish	24.651	0.884	21.299	0.870
	ReLU	**24.658**	**0.886**	**21.361**	**0.879**

**Table 3:** Quantitative analysis of different state-of-the-art LLIE methods on public paired benchmarks. Red/green text means the best/second-best performance. ↓ and ↑ respectively represent the smaller or bigger the value, the better the performance.

Method	LOL				MIT-Adobe			
	MSE ↓	PSNR ↑	SSIM ↑	LPIPS ↓	MSE ↓	PSNR ↑	SSIM ↑	LPIPS ↓
LR3M [18]	4.928	16.998	0.301	0.580	4.117	17.917	0.699	0.241
SRIE [19]	4.902	17.162	0.235	0.552	4.206	17.819	0.690	0.249
BIMEF [20]	4.869	17.191	0.264	0.560	4.259	17.772	0.683	0.252
RetinexNet [54]	1.651	21.875	0.462	0.474	4.391	17.624	0.671	0.239
DSLR [49]	3.536	18.580	0.597	0.337	1.947	21.172	0.692	0.201
KinD [28]	1.431	22.509	0.766	0.143	2.675	19.908	0.799	0.167
DLN [14]	1.515	21.939	0.807	0.163	1.897	16.995	0.769	0.171
DRBN [59]	2.259	20.635	0.472	0.316	3.307	18.875	0.378	0.291
EnlightenGAN [29]	1.998	21.263	0.677	0.322	3.628	18.456	0.745	0.170
MIRNet [65]	1.226	23.191	0.816	0.253	1.864	21.361	0.690	0.238
Zero DCE++ [1]	3.300	14.859	0.587	0.360	3.481	13.197	0.704	0.212
SCL-LLE [52]	2.445	20.197	0.695	0.386	3.002	19.291	0.636	0.279
Ours	1.103	24.658	0.866	0.140	1.412	21.361	0.879	0.169

**Table 4:** Quantitative analysis of different state-of-the-art LLIE methods on public unpaired benchmarks. Red/green text means the best/second-best performance. ↓ and ↑ respectively represent the smaller or bigger the value, the better the performance.

Method	LIME		MEF		NPE		VV	
	NIQE ↓	PCQI ↑	NIQE ↓	PCQI ↑	NIQE ↓	PCQI ↑	NIQE ↓	PCQI ↑
LR3M [18]	4.4259	0.7417	3.6001	0.9459	4.1490	0.7551	3.1233	0.9656
SRIE [19]	3.7870	1.1121	3.5936	0.9570	3.3383	0.9556	3.1361	0.9629
BIMEF [20]	3.8313	1.0647	3.5674	0.9293	3.4027	0.9116	3.1175	0.9271
RetinexNet [54]	4.9079	0.7947	3.7337	0.9112	4.2111	0.7320	3.2440	0.9163
DSLR [49]	5.8877	0.7286	4.1052	0.8998	4.2655	0.7802	3.6661	0.8116
KinD [28]	4.7619	0.9393	3.5954	0.9081	3.5060	0.8638	3.3689	0.8314
DLN [14]	3.8432	0.9990	3.5608	0.9002	3.4119	0.9036	3.1096	0.9292
DRBN [59]	3.8710	1.0059	3.5711	0.9225	3.5413	0.9201	3.2210	9.9199
EnlightenGAN [29]	4.6320	0.9392	3.2232	0.9691	3.5885	0.8897	2.5814	0.9774
Zero DCE++ [1]	3.7691	1.0956	3.5279	0.9398	3.2819	0.9598	2.4001	0.9799
SCL-LLE [52]	3.7800	0.7874	3.3115	0.8991	3.8776	0.7543	3.1649	0.9010
Ours	3.5996	1.2351	3.0010	0.9689	2.9998	0.9696	2.3369	0.9801

**Table 5:** Computational complexity comparison with state-of-the-art methods on LOL benchmark. ↓ means the smaller the value, the better the performance.

Method	Param (M) ↓	Flops (G) ↓	Time (s) ↓
LR3M [18]	-	-	7.4802
SRIE [19]	-	-	5.1453
BIMEF [20]	-	-	0.5096
RetinexNet [54]	1.23	6.79	0.5217
DSLR [49]	14.31	22.95	0.0201
KinD [28]	8.49	7.44	0.6445
DLN [14]	91.19	198.56	0.9807
DRBN [59]	0.58	2.62	0.0711
EnlightenGAN [29]	8.64	7.88	0.6501
Zero DCE++ [1]	1.25 $\times \;10^{-6}$	0.12	0.0028
SCL-LLE [52]	0.08	1.56	0.0048
Ours	2.91	3.08	0.0213

## Data Availability

Publicly available datasets were analyzed in this study. These data can be found here: LOL benchmark https://daooshee.github.io/BMVC2018website/, MIT Adobe FiveK benchmark https://data.csail.mit.edu/graphics/fivek/, and LIME, MEF, NPE, and VV https://drive.google.com/drive/folders/1lp6m5JE3kf3M66Dicbx5wSnvhxt90V4T.
